# Additional Value of 18F-FDOPA Amino Acid Analog Radiotracer to Irradiation Planning Process of Patients With Glioblastoma Multiforme

**DOI:** 10.3389/fonc.2021.699360

**Published:** 2021-07-06

**Authors:** David Sipos, Zoltan László, Zoltan Tóth, Peter Kovács, Jozsef Tollár, Akos Gulybán, Ferenc Lakosi, Imre Repa, Arpad Kovács

**Affiliations:** ^1^ Dr. József Baka Diagnostic, Radiation Oncology, Research and Teaching Center, “Moritz Kaposi” Teaching Hospital, Kaposvár, Hungary; ^2^ Doctoral School of Health Sciences, University of Pécs, Pécs, Hungary; ^3^ Department of Medical Imaging, Faculty of Health Sciences, University of Pécs, Pécs, Hungary; ^4^ MEDICOPUS Healthcare Provider and Public Nonprofit Ltd., Somogy County Moritz Kaposi Teaching Hospital, Kaposvár, Hungary; ^5^ Department of Neurology, Somogy County Moritz Kaposi Teaching Hospital, Kaposvár, Hungary; ^6^ Medical Physics Department, Institut Jules Bordet, Bruxelles, Belgium; ^7^ Department of Oncoradiology, Faculty of Medicine, University of Debrecen, Debrecen, Hungary

**Keywords:** 3,4-dihydroxy-6- [18F] fluoro-l-phenylalanine, 18F-FDOPA, positron emission tomography–computed tomography, positron emission tomography–magnetic resonance, glioblastoma, irradiation, treatment, metabolic activity, target definition

## Abstract

**Purpose:**

To investigate the added value of 6-(18F]-fluoro-L-3,4-dihydroxyphenylalanine (FDOPA) PET to radiotherapy planning in glioblastoma multiforme (GBM).

**Methods:**

From September 2017 to December 2020, 17 patients with GBM received external beam radiotherapy up to 60 Gy with concurrent and adjuvant temozolamide. Target volume delineations followed the European guideline with a 2-cm safety margin clinical target volume (CTV) around the contrast-enhanced lesion+resection cavity on MRI gross tumor volume (GTV). All patients had FDOPA hybrid PET/MRI followed by PET/CT before radiotherapy planning. PET segmentation followed international recommendation: T/N 1.7 (BTV1.7) and T/N 2 (BTV2.0) SUV thresholds were used for biological target volume (BTV) delineation. For GTV-BTVs agreements, 95% of the Hausdorff distance (HD95%) from GTV to the BTVs were calculated, additionally, BTV portions outside of the GTV and coverage by the 95% isodose contours were also determined. In case of recurrence, the latest MR images were co-registered to planning CT to evaluate its location relative to BTVs and 95% isodose contours.

**Results:**

Average (range) GTV, BTV1.7, and BTV2.0 were 46.58 (6–182.5), 68.68 (9.6–204.1), 42.89 (3.8–147.6) cm^3^, respectively. HD95% from GTV were 15.5 mm (7.9–30.7 mm) and 10.5 mm (4.3–21.4 mm) for BTV1.7 and BTV2.0, respectively. Based on volumetric assessment, 58.8% (28–100%) of BTV1.7 and 45.7% of BTV2.0 (14-100%) were outside of the standard GTV, still all BTVs were encompassed by the 95% dose. All recurrences were confirmed by follow-up imaging, all occurred within PTV, with an additional outfield recurrence in a single case, which was not DOPA-positive at the beginning of treatment. Good correlation was found between the mean and median values of PET/CT and PET/MRI segmented volumes relative to corresponding brain-accumulated enhancement (r = 0.75; r = 0.72).

**Conclusion:**

^18F^FDOPA PET resulted in substantial larger tumor volumes compared to MRI; however, its added value is unclear as vast majority of recurrences occurred within the prescribed dose level. Use of PET/CT signals proved to be feasible in the absence of direct segmentation possibilities of PET/MR in TPS. The added value of ^18F^FDOPA may be better exploited in the context of integrated dose escalation.

## Introduction

Glioblastoma multiforme (GBM) is the most aggressive type of central nervous system malignancy. GBM represents 15% of all brain tumors with an incidence of 3/100 000 people. Treatment usually involves surgery, after which chemotherapy and radiation therapy are used. Despite modern treatment methods, GBM usually recurs. The typical duration of survival is 12 to 15 months, and unfortunately, fewer than 7% of the patients survives more than 5 years (1, 2).

In the modern radiotherapy of brain malignancies, the irradiated treatment volumes are based on conventional computed tomography (CT) and magnetic resonance (MR) information. For the macroscopic target volume (gross tumor volume GTV) definition pre- and postoperative gadolinium enhanced T1; and T2-weighted MR images are used, the resection cavity must be considered also if present (2, 3).

The phenomenon of the gadolinium enhancement is based on the disruption of blood-brain barrier, which leads up to difficult determination between postsurgical changes and residual tumor (4, 5). Contrast enhancement may not refer to exact tumor extension, furthermore based on recurrence pattern and tumor infiltration, clinical target volume (CTV) is defined as a 2-cm expansion of the GTV volume for microscopic spread reduced to anatomical barriers (2, 6, 7).

Nowadays, besides the routine target definition process for cerebral tumors, high attention is paid for the integration of positron emission tomography (PET) imaging, which describes biological and functional morphology [biological tumor volume (BTV)], whereas MRI has limited value to identifying the physical effect of the tumor, such as BBB breakdown and edema (8, 9). This additional information can be used for treatment response, noninvasive grading, differential diagnosis, delineation of tumor extent to improve tumor localization, and radiation therapy treatment planning as well (10–12).

In contrast to glucose metabolism of 2-deoxy-2-(fluorine-18)fluoro-d-glucose (18F-FDG), amino acid analog positron emission tomography (PET) radiotracers are characterized by increased accumulation in tumor tissues and low uptake in normal brain tissues (13).

3,4-dihydroxy-6-(18F) fluoro-l-phenylalanine (18F-FDOPA) is one of the most studied amino acid analog radiotracers to describe central nervous system malignancies. As mentioned, radiotracer increased amino acid transport of the tumor cells are responsible for the elevated accumulation at the malignant tissue (3, 14–18).

In the contouring process, a single observer rater provides data, which can suffer from intra- and inter-rater variances (19). For different tumor types and tracers, standardized recommendations were described by the European Association of Nuclear Medicine (EANM), European Association of Neurooncology (EANO), Response Assessment in Neurooncology (RANO) practice guidelines Society of Nuclear Medicine and Molecular Imaging (SNMMI) procedure standards for imaging gliomas (20, 21).

Regarding the current report of the PET/RANO group, the majority of the available data connected to contribution of PET imaging to radiotherapy planning and monitoring are based on studies with (11C-methyl)-l-methionine (MET) and O-[2-(18F)-fluoroethyl]-l-tyrosine FET. During radiotherapy target delineation, MET, FET, and FDOPA studies suggested that BTV characterized by mentioned radiotracers is larger than contrast enhancement in World Health Organization (WHO) grade III/IV gliomas (22–26).

The objective of this study was to compare GTV volume on MRI with the volume of 18F-FDOPA with different segmentation threshold. The signal intensities of 18F-FDOPA PET information by PET-MR and PET-CT were compared regarding relative brain signal. We also studied the target coverage of 18F-FDOPA volume by standard of care approach. We analyzed the location of recurrences relative to MRI-based GTV, PET-based BTV volume, and standard of care PTV volume. We determined the volume of recurrence, if present, to compare to the initial PET BTV thresholds accumulation volume and PTV.

## Materials and Methods

At our institution from September 2017 to December 2020, 17 patients with pathologically confirmed WHO grade IV glioma underwent CT-MR–based radiotherapy up to 60 Gy with VMAT (volumetric arc therapy) plus te-mozolomide (TMZ) per protocol. We excluded from our study those patients who were under treatment for Parkinson’s disease or had contraindications to MRI contrast agent or radiotherapy.

18F-FDOPA radiotracer was produced with an on-site cyclotron (Siemens Eclipse) on the day of the acquisition. The studies were performed using PET/MRI equipment (Siemens Biographs 3.0 T nMR, Erlangen, Deutschland) 10 min after intravenous injection of the radiotracer. Joint EANM/EANO/RANO practice guidelines/SNMMI procedure standards for imaging of gliomas using PET with radiolabelled amino acids and 18F-FDG: version 1.0 recommended using bone segmentation containing MRAC methods. Simultaneous photon emission data collection was performed using one bed position during 30 min. After performing 18F-FDOPA PET/MRI, planning PET/CT (Siemens Biograph Truepoint 64 PET/CT, Erlangen, Deutschland) was performed according to irradiation position protocol, lastly 18F-FDOPA data from PET/CT were co-registrated with planning CT and MRI measurements using rigid registration considering the bony parts of the skull.

Target volumes of GTV and BTV on co-registrated images were described according to the recommendation of the European Organization for Research and Treatment of Cancer (EORTC) by experienced oncoradiologists using Varian Eclipse 13.0 version software (Varian Medical Systems Inc., Palo Alto, CA, USA). CTV was defined as a 2-cm expansion of GTV in proportion of anatomical burdens. Planning target volume (PTV) was defined as 3- to 5-mm additional margin to CTV.

In their study, Patel et al. showed the best differentiation between LG and HGG at the T/N SUVmax ratio greater than 1.7. Based on Patel et al.’s research and EANM/EANO/RANO practice guide-lines/SNMMI procedure standards for imaging of gliomas using PET with radiolabeled amino acids, version 1.0.; T/N 1.7 (in the following BTV 1.7) and T/N 2.0 (in the following BTV 2.0) ratio seemed to be able to deliver the best determination of tumor extend of high-grade gliomas. A 1-cm diameter spherical region of interest (ROI) was placed at the suspected tumor site (T) and contralateral white matter at the level of centrum semiovale to calculate the metabolic activity of the radiotracer using the standard body weight method (27). After calculating the ROI’s activity, BTV 1.7 and BTV 2.0 ratio’s volume coverage was measured. Since the basal ganglia have significant 18F-FDOPA radiotracer uptake, anatomical correction needed to be done to describe basal ganglia region as not a malignant tissue at the cerebral area.

Because of the physical half-life of the 18F-labeled radiotracers, signal intensity deviations were calculated from acquisition time from DICOM header of each PET/MRI and PET/CT to relative brain signal, respectively.

If recurrence was detected on follow-up MRI examinations, the area of recurrence was also contoured and compared with the PTV information.

Institutional ethics committee approved the retrospective analysis of the images (license number: IG/04865-001/2020).

### Statistical Analysis

95% Hausdorff distance from the GTV as reference were considered, whereas supplementary volume contour (SVC) was also calculated to identify the added value of 18F-FDOPA–based BTV segmentation. SVC was measured for each patient to establish the maximum and mean distance between GTV volume and BTV 1.7 and BTV 2.0 volume using Python programming software. Since Varian Eclipse 13.0 is unable to process the PET information from PET/MR directly, we used the Medical Interactive Creative Environment (MICE Toolkit™, version 1.0.6, NONPI Medical AB, Stockholm, Sweden) to calculate linear regression value whether the 18F-FDOPA accumulation ratios are the same at PET/MR and PET/CT imaging modalities regarding normalized brain signal.

## Results

Male patients dominated the sample with the median age of 56.3 years (youngest, 33 years; oldest, 77 years). Biopsy sampling was performed in most patients; all of the study participants had pathologically confirmed WHO grade IV. glioma (**Table 1**).

**Table 1:** Patient and tumor characteristics.

	(n=)
**No. of patients**	17
**Sex**	
Female	5
Male	12
**Age (median, range)**	56.2 (35-77) years
**Histology**	
Glioblastoma (HGG grade-IV)	17
**Extent of resection**	
Biopsy	11
Partial resection	1
Total resection	5

After defining MRI T1CE GTV, BTV 1.7 and BTV 2.0 volumes within GTV and outside GTV were calculated. The tumor extends characteristics are shown in **Table 2**.

**Table 2:** The MRI T1CE GTV; BTV 1.7 and BTV 2.0 and the relation between measured volume (overlap/difference) values are shown in cm^3^.

Patient no.	MRI T1CE volume	BTV 1.7 volume			BTV 2.0 volume		
	GTV	BTV 1.7	Within GTV	Outside GTV	BTV 2.0	Within GTV	Outside GTV
**1.**	29.2	12.4	6.4	5	3.8	2.9	0.5
**2.**	26.8	21.3	9.3	10.3	12.4	7	4
**3.**	73.2	96.7	27.5	68.1	59.6	24.7	33.3
**4.**	19.9	30.7	5.8	24.7	19.7	5.6	13.8
**5.**	55.5	47.3	32.8	10.9	17.8	15.1	1.2
**6.**	188.5	147.6	90.5	51.2	108.8	76.2	27.3
**7.**	57.1	29.8	15.4	12.9	16.1	9.9	17.7
**8.**	66.7	20.1	8.4	10.3	10.4	6	3
**9.**	117.7	47.8	11	35.4	12.6	2.4	8.7
**10.**	66.9	40.1	17.9	19.8	27.3	14	11.4
**11.**	21.3	57.4	22.4	3.1	36	20.1	4.9
**12.**	69.1	69.3	30.9	31.8	35.7	23.2	6.4
**13.**	158.9	196	139.7	38.1	147.6	117.8	19.2
**14.**	175.1	204.1	53.7	146.8	129.5	49.4	76.9
**15.**	30.1	9.6	2.4	5.9	6.1	1.5	3.6
**16.**	7.9	11	3.9	5.8	2.8	2	0.3
**17.**	10.3	2	0	1.9	0	0	0

**Figure 1:**
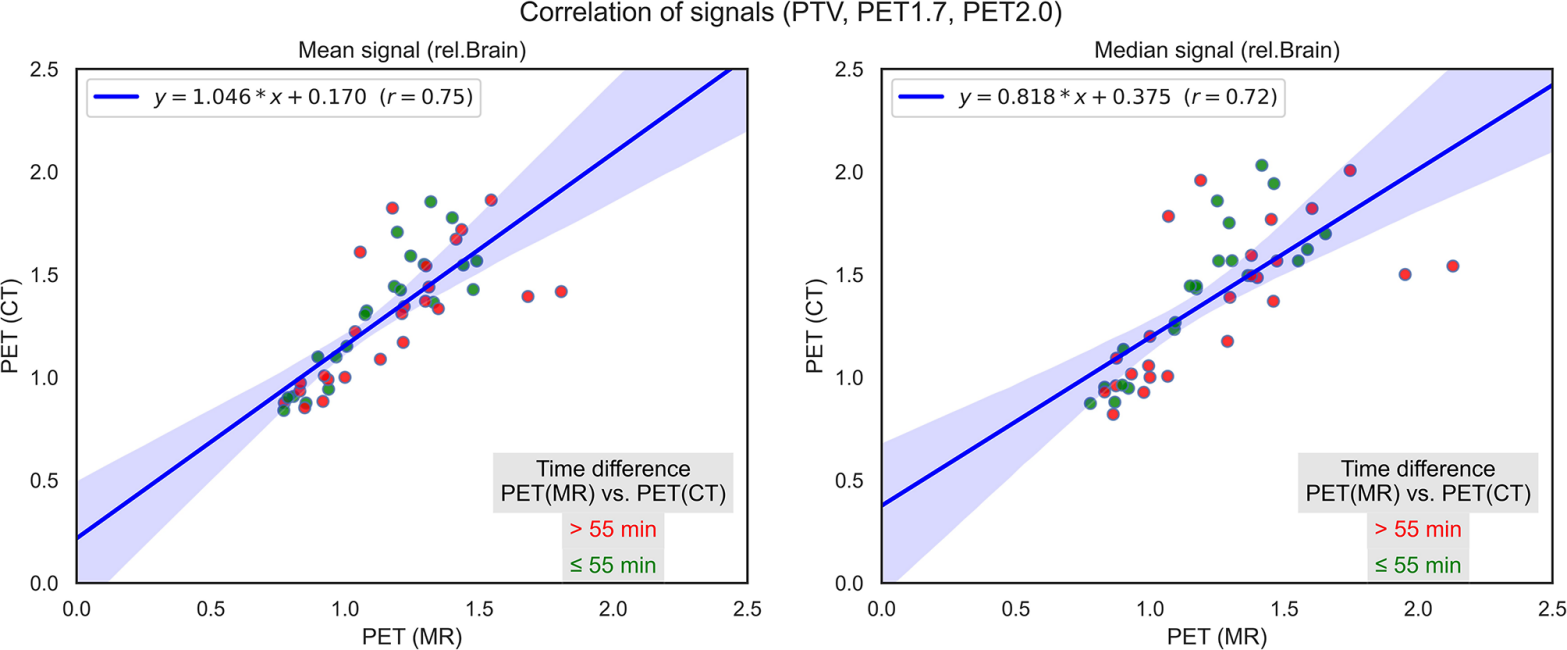
Correlation of PET/CT and PET/MR signals regarding PTV, 18F-FDOPA T/N 1.7, and 18F-FDOPA T/N 2.0 volume indicating the time difference between the two acquisitions.

PET/CT and PET/MR compared to the relative signal values of PET/CT and PET/MR. A good correlation value can be found between the mean and median values of the variables (r=0.75; r=0.72) (**Figure 1**).

**Figure 2:**
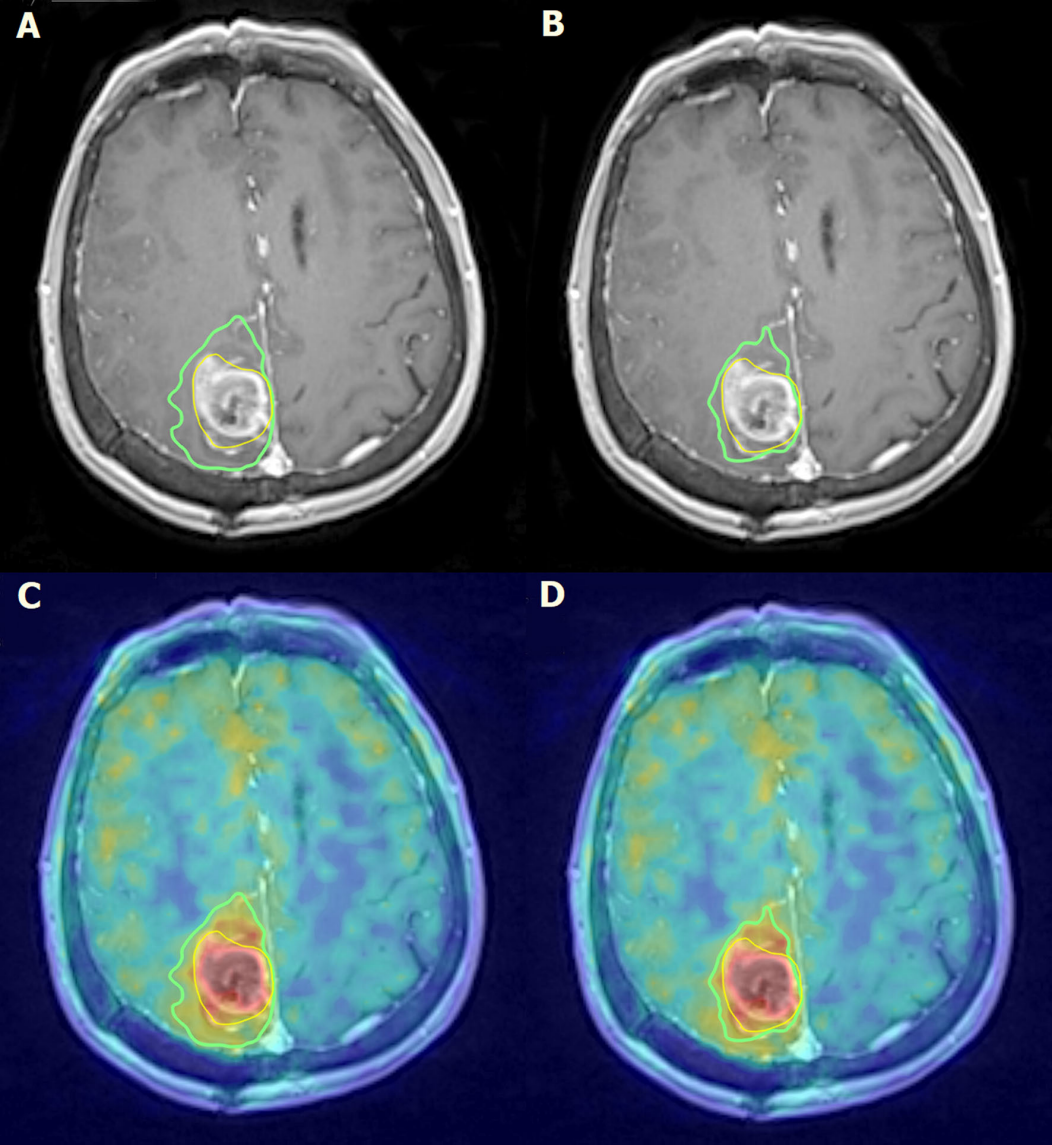
Parieto-medial postoperative status on the right side of the brain. In the surgical region, a fluid-containing cavity with small air bubbles and uneven contrast enhancement can be detected on post-contrast T1-weighted MRI images **(A, B)**. Uneven 18F-FDOPA accumulation can be observed at the edges of the deviation on fused 18F-FDOPA PET/MRI images **(C, D)**. Intense contrast and 18F-FDOPA accumulation suggests of the presence of a residual tumor around the surgical cavity. The yellow line describes the GTV volume **(A–D)**, the green line on panels **(A, C)** describes the BTV 1.7 accumulation coverage and the green line on panels **(B, D)** describes the BTV 2.0 accumulation coverage.

**Figure 3:**
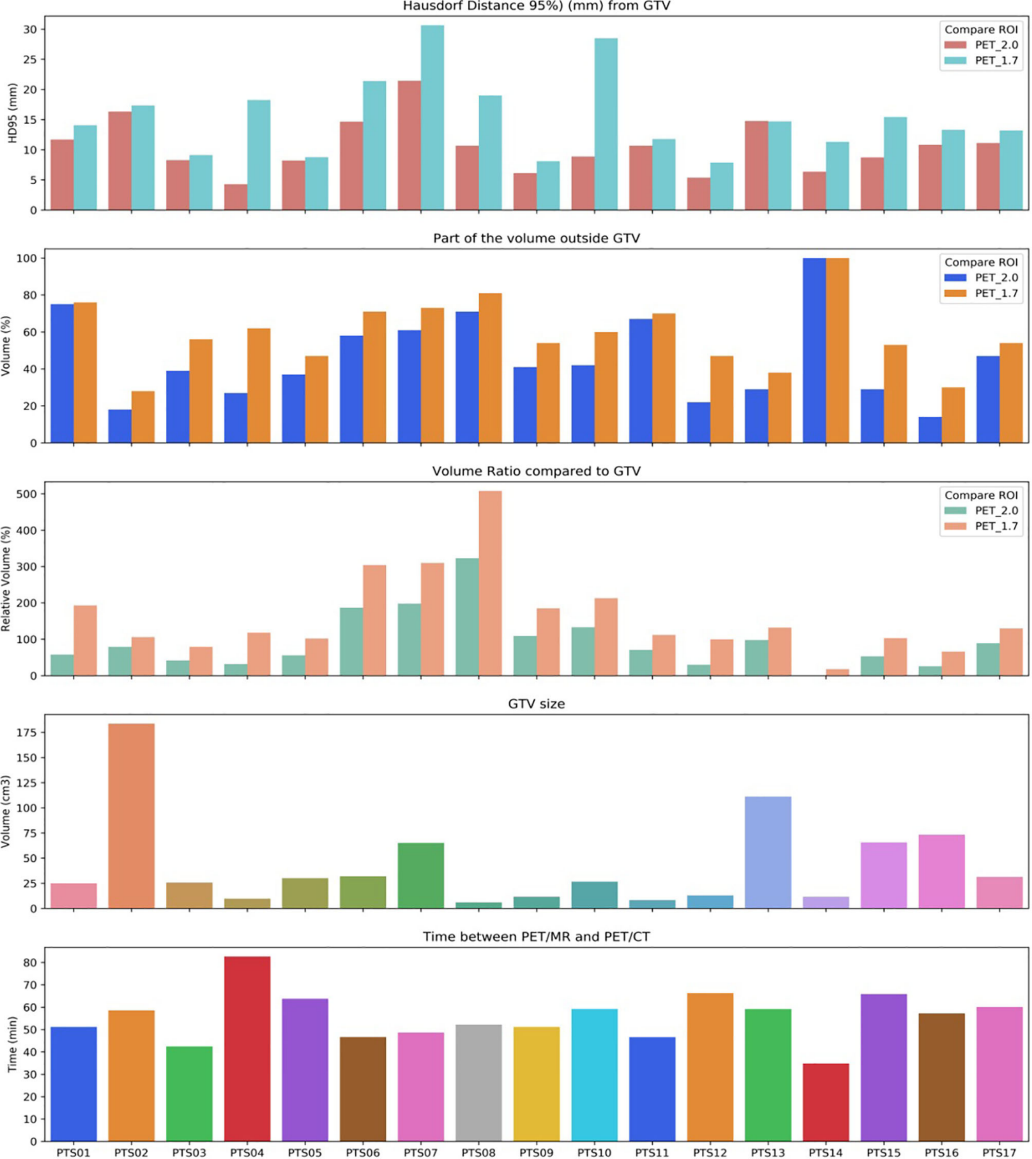
Calculated Hausdorff distance and GTV volume ratios compared to BTV 1.7 and BTV 2.0 volumes and relative GTV size are shown. Time elapsed between PET/MRI and PET/CT scans was also indicated per patient.

On average, 95% of the segmented volumes were within 15.5 mm (range, 7.9–30.7 mm) and 10.5 mm (range, 4.3–21.4 mm) for BTV 1.7 and BTV 2.0 respectively. For the disagreement, we used the percentage of the volume outside of the reference (in this case GTV) for BTV 1.7, on average, 58.8% (range, 28–100%) were outside the GTV, whereas this lowered to 45.7% (range, 14–100%) regarding BTV 2.0. Both PET volumes remained within the adequate dose coverage (95% of the prescribed dose), only on a few cases had minor coverage loss up to maximum 3% and 5% of their volume for BTV 2.0 and BTV 1.7, respectively. GTV varied substantially within our cohort, with an average 43 cm^3^ (range, 6.1–183.6 cm^3^) (**Figure 2**). Time between the PET/MR and PET/CT acquisition varied between 35 and 83 min, with an average around 1 h (55.7 min) (**Figure 3**).

**Figure 4:**
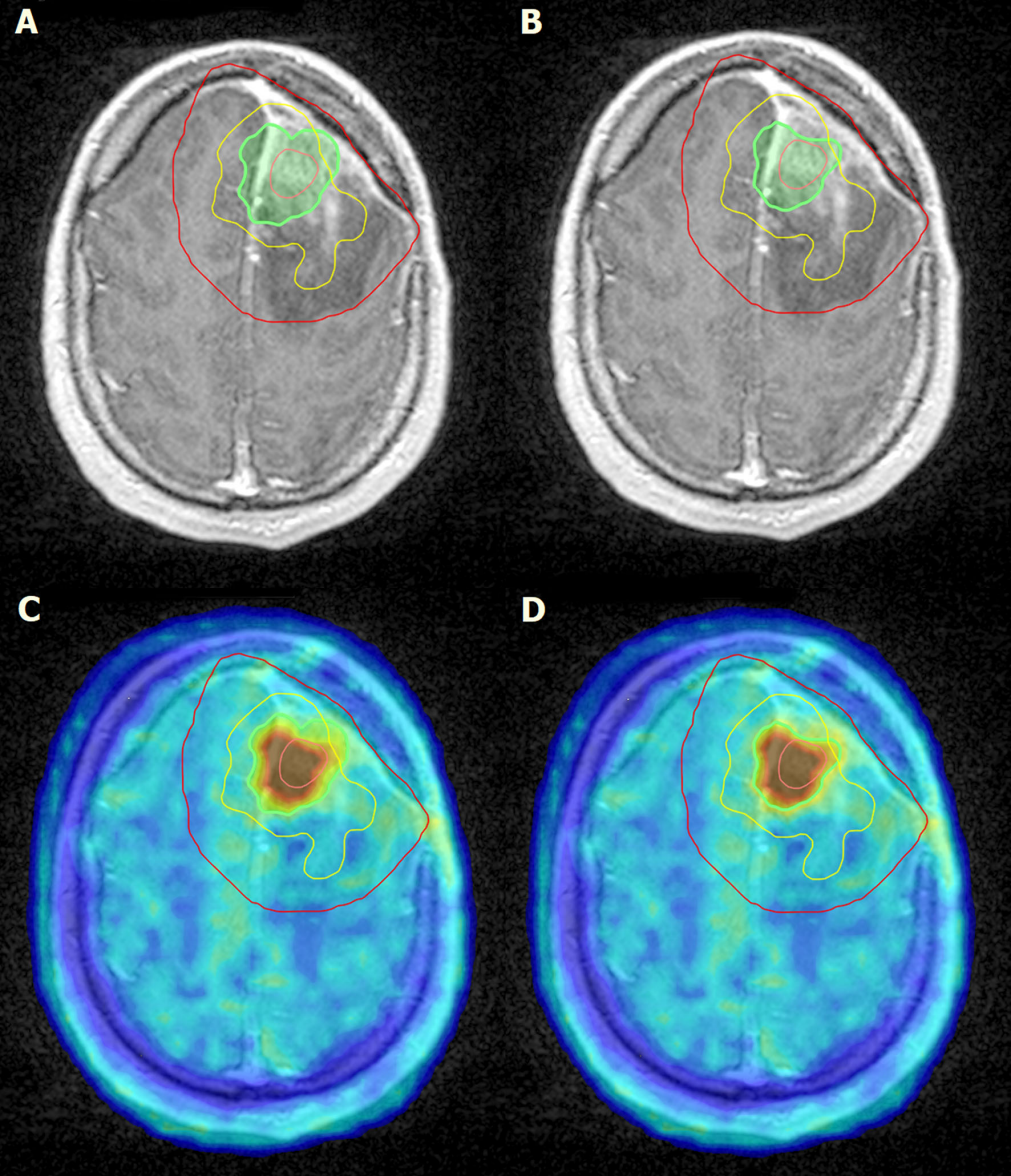
Left frontal post craniotomy status. Inhomogeneous, mainly centrally, moderate enhancement of contrast material is observed on T1-weighted post contrast MRI images. The lesion in the left hemisphere is surrounded by edema **(A, B)**. Irregularly shaped intense, focal 18F-FDOPA accumulation can be detected on the left side of the brain frontally, above the level of lateral ventricles **(C, D)**. Pink line, GTV; green line, BTV 1.7 **(A, C)**; green line, BTV 2.0 **(B, D)**; red line, PTV; yellow line, recurrence.

During patient follow-up, recurrence was detected in four cases. 68% (range, 9–94%) of the recurrence was detected outside the GTV volumes. Regarding BTV 1.7 and BTV 2.0, on average, 67% (range, 39–81) and 79% (range, 69–89) of the recurrence occurred outside BTV volumes, respectively (**Figure 4**). There was a negligible volume recurrence outside the PTV area (average, 0.01%; range, 0.01–0.05%).

## Discussion

Our research focused on the potential additional information of 18F-FDOPA during the irradiation planning process of patients with glioblastoma multiforme. We illustrated the BTV 1.7 and BTV 2.0 volume coverage to GTV described by neurooncologists according to valid recommendation regarding high-grade gliomas. We also examined the area of recurrence in proportion to PTV.

Because of its nature (high degree of vascularity, rapidly dividing cells, invasion into normal brain tissue), the treatment of glioblastoma multiforme is a complex oncology task, which remains a serious challenge despite today’s modern technology; needless to say, overall survival is still unsatisfactory (28). Standard therapy includes surgical resection to the extent feasible and radiotherapy followed with concomitant and adjuvant chemotherapy (29). Conventional imaging modalities provide information regarding the anatomical distribution, whereas PET imaging displays molecular information about malignant abnormalities. The combination of these modalities play a key role at the standard care of management at central nervous system malignancies for surgical purposes, radiation planning, and treatment assessment as well (11, 30).

18F-FDG was the first radiotracer used to diagnose brain tumors, which provided useful information for differentiation WHO grade III/IV gliomas but its specificity seemed to be limited because of high normal brain tissue accumulation. Furthermore, in brain abscesses, demyelinating tumefactive lesions can be described with higher 18F-FDG metabolism, as well as malignant tissues (31).

In 2016, the Response Assessment in Neuro-Oncology (RANO) working group and European Association for Neuro-Oncology (EANO) highlighted the role of amino acid PET as a particularly important imaging tool for central nervous system malignancies especially when determining the tumor burden. As previously mentioned, in contrast of 18F-FDG, amino acid analog tracers have high accumulation in malignant lesions and relatively low accumulation in normal brain tissue; furthermore, they have the ability to pass through blood-brain barrier without disruption. The most studied researches among amino acid radiotracers focused on 11C-MET, 18F-FET, and 18F-FDOPA. The physical half-life of 11C (20 min) is significantly lower than 18F (109 min) that is the reason why 11C-MET is not widely used in clinical routine unless the institution has onsite cyclotron (32–35).

As a radiolabeled dopamine precursor 18F-FDOPA was initially used in Parkinson’s disease diagnostics since the 1980s. The first case report about potential neurooncologic application was published in 1996, a patient with movement disorder underwent 18F-DOPA PET examination and besides asymmetrically reduced dopamine uptake in the putamen, the imaging revealed incidental focal pathologic tracer uptake in other cerebral area, and MR and surgical histology confirmed glioma as an underlying pathology. The case study provided evidence that 18F-DOPA PET could also be suitable for the evaluation of central nervous system malignancies; however, because of the high physiologic DOPA uptake, abnormalities located near or involving the striatum can be challenging to evaluate. Nowadays, besides being a primer diagnosis, 18F-FDOPA is used for detection recurrence, in grading, to predict survival, irradiation planning, and for detection brain metastasizes as well (36–38).

The use of amino acid tracers, such as 18F-FDOPA, enables to represent tumor components beyond contrast enhancement of T1CE MRI images (39). A study performed by Pafundi et al. showed that 18F-FDOPA SUVmax has the ability to distinguish low-grade and high-grade lesions. Therefore, using SUV-based stereotactic biopsy selection and definition of high-grade areas of malignant volume may be valuable added information when delineating radiotherapy boost volumes (20). We need to mention that other retrospective studies confirmed that larger PTVs failed to produce significant reduction of recurrences at close tumor area and even at distant recurrences also led to an increased incidence of radiation-induced necrosis/toxicity, which affected negatively on patients’ long-term survival (40, 41).

According Kazda et al., the T1-CE MRI-based GTV volumes increased considering the 18F-FDOPA information, which did not result in increased doses of the organs at risk. Dowson et al. concluded that volumes defined by 18F-FDOPA PET resulted in larger GTV as well than T1-CE MRI-based GTV. Therefore, MRI and 18F-FDOPA PET-based treatment planning appeared feasible in patients with high-grade gliomas (42, 43).

Another study by Kosztyla et al. stated that uniform dose escalation at high-grade gliomas resulted unfavorable outcome results regarding non-central relapses. According to their result, 18F-FDOPA accumulation of malignant tissues proceeded to larger burden of disease; therefore, dose painting may contribute to better disease control. Their study suggested that dose painting of dedicated treatment volume is feasible while the dose of the organs at risk remain the same (44).

As we described previously, PET/MR information cannot be directly processed into Varian Eclipse 13.0 software. Because the PET/MR was performed before PET/CT acquisition, we needed to validate whether the PET intensity from PET/MR correlates to PET information gained from PET/CT or not. The MICE Toolkit™, a graphical programming user interface that is user-friendly while still highly flexible, selected DICOM images from PET/MRI and PET/CT, which were co-registered, and the volumes contoured on patients’ data were also examined to gain correlation values. As a result of our method, good correlation values between PET/MR and PET/CT 18F-FDOPA signal intensity to relative brain signal were gained. This result means that the measurement of the contralateral normal-appearing white matter is a good way to determine 18F-FDOPA uptake value to establish tumor burden and use of the MICE Toolkit™ is a highly recommended for method validation.

The obtained BTV values differed from the traditionally defined GTV values. Almost 60% of BTV 1.7 volume coverage was outside the GTV area, and almost 50% of the BTV 2.0 volume coverage was outside the GTV area, respectively. Outside GTV volumes carry additional information regarding conventional imaging methods (CT, MR). Because the CTV area is an extension of the GTV, any information beyond the traditionally defined GTV can modify PTV. Regarding our results, 18F-FDOPA, as an amino acid analog radiotracer, should play a very important role in radiation planning procedure at patients with glioblastoma multiform.

At our study population, recurrence occurred in four patients. We co-registrated the primer 18F-FDOPA PET images with the MRI images where the recurrences were present to see whether the recurrence is overlapping with the PTV area, the area which received the total dose of 60 Gy. At all cases recurrence occurred mostly under PTV area, no outfield recurrence was detected.

Weber et al. examined the 18F-FET radiotracer’s additional value compared 19 patients’ GTVs and CTVs. They found that BTVs were substantially larger than their morphologic counterpart, but paradoxically, this deviation did not resulted significant increase of the target fields during RT planning (22). Piroth et al. analyzed the relapse patterns of 13 patients using 18F-FET-PET and MRI based integrated-boost intensity-modulated radiochemotherapy. The location of the recurrence was analyzed and related to the initial tumor detected in baseline FET-1 acquisition. According to them, the contrast-enhanced MRI does not reliably reflect the extent of the FET uptake neither on baseline and nor on recurrence acquisitions, the relapse pattern was only 13% in median only. Just like at our study population, 100% of the tumor recurrences were located under MRI based, routinely performed target volumes achieving 60 Gy. More than two thirds of tumor recurrence in FET-PET was located outside the boost volume. Regarding Piroth et al., a CTV based on FET with an extra 7 mm may cover 100% of recurrences, leading to reduced PTV. This volume definition may achieve similar therapeutic level with significantly lower side effects for the patient (23). Niyazi et al. analyzed the re-recurrence in recurrent HGG patients undergoing re-irradiation with bevacizumab at a 31-patient population using 18F-FET PET and MRI. Regarding their results, the appeared recurrences were mainly located centrally (24). According Lee et al.’s research, the pretreatment 11C-MET-PET uptake region appeared at the highest risk for recurrences at patients with glioblastoma multiforme, which may provide potentially important additional information. They also found significant correlation between the presence of increased MET-PET uptake outside the high-dose region and subsequent non-central failure (25). Referring to PET/RANO group, the most frequently used amino acid analog radiotracers are MET, FET, and FDOPA. In delineation of radiotherapy target volumes, FDOPA may extend beyond the contrast enhancement on MRI. The concept of dose-painting also seems to be feasible and safe in the PET-based radiotherapy with newly diagnosed gliomas. Recent studies confirmed by FDOPA acquisitions showed that acute and late toxicities were not increased in patients who were treated with integrated boost IMRT beyond 60 Gy, but further studies are still ongoing (26).

Although the result of our study suggests that 18F-FDOPA PET-based treatment planning is feasible, future studies should be implemented with a larger patient sample. Currently, MRI imaging is considered as golden standard for glioma RT treatment planning. Several studies suggested that amino acid analog tracers have the ability to detect malignant tumor tissue above CE-T1 MRI area but larger PTV resulted in various side effects for patients. Because of the mentioned additional diagnostic ability of the 18F-FDOPA radiotracer, there is increasing evidence of the usefulness of the amino acid analog-based radiotracers in the planning process of gliomas but the consensus have not been reached yet. The ongoing development of imaging modalities should improve the radiation therapy targeting in which dosimetric analysis may provide more influence to deliver doses more accurately.

## Conclusion

BTV 1.7 volumes were 58.8%; BTV 2.0 volumes were 45.7% outside of the generally described GTV volume. Recurrences occurred below the PTV area which received the full dose of the treatment, no outfield recurrence of the disease appeared. The interpretation of our results is limited by the fact that there are few cases available but the additional value of 18F-FDOPA should be considered when delineating target volumes to improve patient care, optimize outcome, and deliver more focused therapies.

## Data Availability Statement

The original contributions presented in the study are included in the article/supplementary material. Further inquiries can be directed to the corresponding author.

## Ethics Statement

The studies involving human participants were reviewed and approved by Institutional ethics committee (license number: IG/04865-001/2020). The patients/participants provided their written informed consent to participate in this study.

## Author Contributions

DS, FL, ZT, and AK participated in the conceptualization. AG and ZT participated in the methodology. ZL, PK, and JT participated in the investigation. IR participated in the resources. DS participated in the writing—original draft preparation. DS, LF, and AK participated in the writing—review and editing. All authors contributed to the article and approved the submitted version.

## Funding

DS and AK were supported by the New National Excellence Program of the Ministry for Innovation and Technology ÚNKP-18-3-II.

## Conflict of Interest

The authors declare that the research was conducted in the absence of any commercial or financial relationships that could be construed as a potential conflict of interest.

## Abbreviations

GBM, glioblastoma multiforme; CT, computed tomography; MR, magnetic resonance; GTV, gross tumor volume; CTV, clinical target volume; BTV, biological tumor volume; 18F-FDG, 2-deoxy-2-[fluorine-18]fluoro-D-glucose; PET, positron emission tomography; 11C-MET, 11C-methionine; 18F-FET, O- (2- [18F] fluoroethyl) -L-tyrosine; 18F-FDOPA, 3,4-dihydroxy-6- [18F] fluoro-L-phenylalanine; EANM, European association of nuclear medicine; EANO, European association of neurooncology; RANO, response assessment in neurooncology; SNMMI, society of nuclear medicine and molecular imaging; WHO, World Health Organization; VMAT, volumetric arc therapy; EORTC, European Organization for Research and Treatment of Cancer; Gy, gray; PTV, planning target volume; TMZ, temozolomide; SUVmax, standardized uptake value.

## References

[1] Lukács G Tóth Z Sipos D Csima M Hadjiev J Bajzik G. Long-Term Follow-Up Results of Concomitant Chemoradiotherapy Followed by Adjuvant Temozolomide Therapy for Glioblastoma Multiforme Patients: The Importance of MRI Information in Survival: Single-center Experience. . Ideggyogy sz (2018) 71:95–103. 10.18071/isz710095 29889468

[2] Niyazi M Brada M Chalmers AJ Combs SE Erridge SC Fiorentino A. Estro-ACROP Guideline “Target Delineation of Glioblastomas”. Radiother Oncol (2016) 118:35–42. 10.1016/jradonc201512003 26777122

[3] Somme F Bender L Namer IJ Noël G Bund C. Usefulness Of 18F-FDOPA PET for the Management of Primary Brain Tumors: A Systematic Review of the Literature. Cancer Imaging (2020) 20:70. 10.1186/s40644-020-00348-5 33023662 PMC7541204

[4] Mrowczynski OD Zammar S Bourcier AJ Langan ST Liao J Specht CS. Utility of Early Postoperative Magnetic Resonance Imaging After Glioblastoma Resection: Implications on Patient Survival. World Neurosurg (2018) 120:e1171–4. 10.1016/jwneu201809027 30218799

[5] Belhawi SM Hoefnagels FW Baaijen JC Aliaga ES Reijneveld JC Heimans JJ. Early Postoperative MRI Overestimates Residual Tumour After Resection of Gliomas With No or Minimal Enhancement. Eur Radiol (2011) 21:1526–34. 10.1007/s00330-011-2081-y PMC3101346 21331595

[6] Fathi Kazerooni A Nabil M Zeinali Zadeh M Firouznia K Azmoudeh-Ardalan F Frangi AF. Characterization of Active and Infiltrative Tumorous Subregions From Normal Tissue in Brain Gliomas Using Multiparametric MRI. J Magn Reson Imaging (2018) 48:938–50. 10.1002/jmri25963 PMC6081259 29412496

[7] Harat M Małkowski B Wiatrowska I Makarewicz R Roszkowski K. Relationship Between Glioblastoma Dose Volume Parameters Measured by Dual Time Point Fluoroethylthyrosine-PET and Clinical Outcomes. Front Neurol (2018) 22:756. 10.3389/fneur.2017.00756 PMC5786516 29403428

[8] Hatzoglou V Yang TJ Omuro A Gavrilovic I Ulaner G Rubel J. A Prospective Trial of Dynamic Contrast-Enhanced MRI Perfusion and Fluorine-18 FDG Pet-CT in Differentiating Brain Tumor Progression From Radiation Injury After Cranial Irradiation. Neuro Oncol (2016) 18:873–80. 10.1093/neuonc/nov301 PMC4864262 26688076

[9] Ginet M Zaragori T Marie PY Roch V Gauchotte G Rech F. Integration of Dynamic Parameters in the Analysis Of 18F-Fdopa PET Imaging Improves the Prediction of Molecular Features of Gliomas. Eur J Nucl Med Mol Imaging (2020) 47:1381–90. 10.1007/s00259-019-04509-y 31529264

[10] la Fougère C Suchorska B Bartenstein P Kreth FW Tonn JC. Molecular Imaging of Gliomas With PET: Opportunities and Limitations. Neuro Oncol (2011) 13:806–19. 10.1093/neuonc/nor054 PMC3145468 21757446

[11] Fraioli F Shankar A Hyare H Ferrazzoli V Militano V Samandouras G. The Use of Multiparametric 18F-Fluoro-L-34-Dihydroxy-Phenylalanine PET/MRI in Post-Therapy Assessment of Patients With Gliomas. Nucl Med Commun (2020) 41:517–25. 10.1097/MNM0000000000001184 32282634

[12] Zaragori T Ginet M Marie PY Roch V Grignon R Gauchotte G. Use of Static and Dynamic (18F)-F-DOPA PET Parameters for Detecting Patients With Glioma Recurrence or Progression. EJNMMI Res (2020) 10:56. 10.1186/s13550-020-00645-x 32472232 PMC7260331

[13] Isselbacher KJ. Sugar and Amino Acid Transport by Cells in Culture–Differences Between Normal and Malignant Cells. N Engl J Med (1972) 286:929–33. 10.1056/NEJM197204272861707 4335317

[14] Herholz K Hölzer T Bauer B Schröder R Voges J Ernestus RI. 11C-Methionine PET for Differential Diagnosis of Low-Grade Gliomas. Neurology (1998) 50:1316–22. 10.1212/wnl5051316 9595980

[15] Zaragori T Guedj E Verger A. Is IDH Mutation Status Associated With 18F-Fdopa PET Uptake? Ann Nucl Med (2020) 34:228–9. 10.1007/s12149-020-01442-1 32002736

[16] Wester HJ Herz M Weber W Heiss P Senekowitsch-Schmidtke R Schwaiger M. Synthesis and Radiopharmacology of O-(2-(18F)fluoroethyl)-L-tyrosine for Tumor Imaging. J Nucl Med (1999) 40:205–12. 9935078

[17] Weber WA Wester HJ Grosu AL Herz M Dzewas B Feldmann HJ. O-(2-(18F)Fluoroethyl)-L-tyrosine and L-(methyl-11C)methionine Uptake in Brain Tumours: Initial Results of a Comparative Study. Eur J Nucl Med (2000) 27:542–9. 10.1007/s002590050541 10853810

[18] Wahl L Nahmias C. Modeling of Fluorine-18-6-fluoro-L-Dopa in Humans. J Nucl Med (1996) 37:432–7. 8772639

[19] Deeley MA Chen A Datteri R Noble JH Cmelak AJ Donnelly EF. Comparison of Manual and Automatic Segmentation Methods for Brain Structures in the Presence of Space-Occupying Lesions: A Multi-Expert Study. Phys Med Biol (2011) 56:4557–77. 10.1088/0031-9155/56/14/021 PMC3153124 21725140

[20] Pafundi DH Laack NN Youland RS Parney IF Lowe VJ Giannini C. Biopsy Validation of 18F-DOPA PET and Biodistribution in Gliomas for Neurosurgical Planning and Radiotherapy Target Delineation: Results of a Prospective Pilot Study. Neuro Oncol (2013) 15:1058–67. 10.1093/neuonc/not002 PMC3714146 23460322

[21] Law I Albert NL Arbizu J Boellaard R Drzezga A Galldiks N. Joint EANM/EANO/RANO Practice Guidelines/SNMMI Procedure Standards for Imaging of Gliomas Using PET With Radiolabelled Amino Acids and (18F)FDG: Version 10. Eur J Nucl Med Mol Imaging (2019) 46:540–57. 10.1007/s00259-018-4207-9 PMC6351513 30519867

[22] Weber DC Zilli T Buchegger F Casanova N Haller G Rouzaud M. [(18)F]Fluoroethyltyrosine- Positron Emission Tomography-Guided Radiotherapy for High-Grade Glioma. Radiat Oncol (2008) 3:44. 10.1186/1748-717X-3-44 19108742 PMC2628662

[23] Piroth MD Galldiks N Pinkawa M Holy R Stoffels G Ermert J. Relapse Patterns After Radiochemotherapy of Glioblastoma With FET PET-Guided Boost Irradiation and Simulation to Optimize Radiation Target Volume. Radiat Oncol (2016) 11:87. 10.1186/s13014-016-0665-z 27342976 PMC4920983

[24] Niyazi M Jansen NL Rottler M Ganswindt U Belka C. Recurrence Pattern Analysis After Re-Irradiation With Bevacizumab in Recurrent Malignant Glioma Patients. Radiat Oncol (2014) 9:299. 10.1186/s13014-014-0299-y 25529015 PMC4307885

[25] Lee IH Piert M Gomez-Hassan D Junck L Rogers L Hayman J. Association of 11C-Methionine PET Uptake With Site of Failure After Concurrent Temozolomide and Radiation for Primary Glioblastoma Multiforme. Int J Radiat Oncol Biol Phys (2009) 73:479–85. 10.1016/j.ijrobp.2008.04.050 PMC2652133 18834673

[26] Galldiks N Niyazi M Grosu AL Kocher M Langen KJ Law I. Contribution of PET Imaging to Radiotherapy Planning and Monitoring in Glioma Patients - A Report of the PET/RANO Group. Neuro Oncol (2021) 23:881–93. 10.1093/neuonc/noab013 PMC8168815 33538838

[27] Patel CB Fazzari E Chakhoyan A Yao J Raymond C Nguyen H. 18f-Fdopa PET and MRI Characteristics Correlate With Degree of Malignancy and Predict Survival in Treatment-Naïve Gliomas: A Cross-Sectional Study. J Neurooncol (2018) 139:399–409. 10.1007/s11060-018-2877-6 29679199 PMC6092195

[28] Nakada M Furuta T Hayashi Y Minamoto T Hamada J. The Strategy for Enhancing Temozolomide Against Malignant Glioma. Front Oncol (2012) 98:1–5. 10.3389/fonc201200098 PMC3418701 22912934

[29] Stupp R Mason WP van den Bent MJ Weller M Fisher B Taphoorn MJ. Radiotherapy Plus Concomitant and Adjuvant Temozolomide for Glioblastoma. N Eng J Med (2005) 352:987–96. 10.1056/NEJMoa043330 15758009

[30] Lai M Vassallo I Lanz B Poitry-Yamate C Hamou MF Cudalbu C. In Vivo Characterization of Brain Metabolism By 1 H MRS 13 C MRS And 18 FDG PET Reveals Significant Glucose Oxidation of Invasively Growing Glioma Cells. IJC10. (2018) 143:127–38. 10.1002/ijc31299 29417580

[31] Omuro AM Leite CC Mokhtari K Delattre JY. Pitfalls in the Diagnosis of Brain Tumours. Lancet Neurol (2006) 5:937–48. 10.1016/S1474-4422(06)70597-X 17052661

[32] Becherer A Karanikas G Szabó M Zettinig G Asenbaum S Marosi C. Brain Tumour Imaging With PET: A Comparison Between (18F)Fluorodopa and (11C)Methionine. Eur J Nucl Med Mol Imaging (2003) 30:1561–7. 10.1007/s00259-003-1259-1 14579097

[33] Garnett ES Firnau G Nahmias C. Dopamine Visualized in the Basal Ganglia of Living Man. Nature10. (1983) 305:137–8. 10.1038/305137a0 6604227

[34] Leenders KL Frackowiak RS Lees AJ. Steele-Richardson-Olszewski Syndrome Brain Energy Metabolism Blood Flow and Fluorodopa Uptake Measured by Positron Emission Tomography. Brain10 (1988) 111:615–30. 10.1093/brain/1113615 3133078

[35] Youland RS Kitange GJ Peterson TE Pafundi DH Ramiscal JA Pokorny JL. The Role of LAT1 in (18)F-DOPA Uptake in Malignant Gliomas. J Neurooncol (2013) 111:11–8. 10.1007/s11060-012-0986-1 PMC3907171 23086431

[36] Heiss WD Wienhard K Wagner R Lanfermann H Thiel A Herholz K Pietrzyk U. F-Dopa as an Amino Acid Tracer to Detect Brain Tumors. J Nucl Med (1996) 37:1180–2. 8965194

[37] Stegmayr C Stoffels G Filß C Heinzel A Lohmann P Willuweit A. Current Trends in the Use of O-(2-(18F)fluoroethyl)-L-tyrosine ((18F)FET) in Neurooncology. Nucl Med Biol (2020) 92:78–84. 10.1016/jnucmedbio202002006 32113820

[38] Bell C Dowson N Puttick S Gal Y Thomas P Fay M. Increasing Feasibility and Utility of (18)F-FDOPA PET for the Management of Glioma. Nucl Med Biol (2015) 42:788–95. 10.1016/jnucmedbio201506001 26162582

[39] Langen KJ Galldiks N Hattingen E Shah NJ. Advances in Neuro-Oncology Imaging. Nat Rev Neurol (2017) 13:279–89. 10.1038/nrneurol201744 28387340

[40] Minniti G Amelio D Amichetti M Salvati M Muni R Bozzao A. Patterns of Failure and Comparison of Different Target Volume Delineations in Patients With Glioblastoma Treated With Conformal Radiotherapy Plus Concomitant and Adjuvant Temozolomide. Radiother Oncol (2010) 97:377–81. 10.1016/jradonc201008020 20855119

[41] Chang EL Akyurek S Avalos T Rebueno N Spicer C Garcia J. Evaluation of Peritumoral Edema in the Delineation of Radiotherapy Clinical Target Volumes for Glioblastoma. Int J Radiat Oncol Biol Phys (2007) 68:144–50. 10.1016/jijrobp200612009 17306935

[42] Kazda T Pafundi DH Kraling A Bradley T Lowe VJ Brinkmann DH. Dosimetric Impact of Amino Acid Positron Emission Tomography Imaging for Target Delineation in Radiation Treatment Planning for High-Grade Gliomas. Phys Imaging Radiat Oncol (2018) 6:94–100. 10.1016/jphro201806004 33458396 PMC7807641

[43] Dowson N Fay M Thomas P Jeffree R McDowall R Winter C. Contribution of FDOPA PET to Radiotherapy Planning for Advanced Glioma. In J Phys: Conf Series (2014) 489:1. 10.1088/1742-6596/489/1/012028

[44] Kosztyla R Raman S Moiseenko V Reinsberg SA Toyota B Nichol A. Dose-Painted Volumetric Modulated Arc Therapy of High-Grade Glioma Using 34-Dihydroxy-6-(18F)fluoro-L-phenylalanine Positron Emission Tomography. Br J Radiol (2019) 92:20180901. 10.1259/bjr20180901 31017449 PMC6636270

